# Thrombotic thrombocytopenic purpura complicated with acute aortic dissection: A case report

**DOI:** 10.1097/MD.0000000000027898

**Published:** 2021-11-19

**Authors:** Mei-Juan Huang, Jin-Niu Deng, Li-Li Gao, Jian-Feng Zhou

**Affiliations:** Department of Hematology, Tongji Hospital of Tongji Medical College, Huazhong University of Science and Technology, 1095 Jie-Fang Avenue, Wuhan, Hubei, P.R. China.

**Keywords:** acute aortic dissection, connective tissue disease, thoracic aortic endovascular repair, thrombotic thrombocytopenic purpura

## Abstract

**Rationale:**

Thrombotic thrombocytopenic purpura (TTP) is a critical thrombotic microangiopathy involving multiple organs. To the best of our knowledge, there are no reports of TTP complicated by acute aortic dissection.

**Patient concerns:**

We herein described a 53-year-old male with TTP who did not have a significant medical history. After immediate plasma exchange and glucocorticoid therapy, the patient's clinical condition improved. However, the patient suddenly experienced chest pain with elevated blood pressure.

**Diagnoses:**

Computed tomography angiography suggested acute type B aortic dissection.

**Interventions:**

The patient was immediately transferred to the cardiac aortic surgery department for thoracic aortic endovascular repair.

**Outcomes:**

The patient was discharged after successful thoracic aortic endovascular repair. Unfortunately, 3 months later, the patient experienced chest and back pain at home and died suddenly, possibly due to the recurrence of aortic dissection.

**Lessons:**

Even if patients have no identifiable risk factors, physicians should be aware of this rare and life-threatening acute complication of TTP, which may have multiple causes, including preexisting connective tissue disease, abnormal blood pressure fluctuations, and increased risk of hemorrhage. Early identification and timely treatment of acute aortic dissection are critical for improving prognosis.

## 1 Introduction

Thrombotic thrombocytopenic purpura (TTP) is a life-threatening disease with 90% mortality and is characterized by microangiopathic hemolytic anemia, severe thrombocytopenia, neurological abnormalities, fever, and renal insufficiency.^[1]^ Inherited mutations in the ADAMTS13 gene or autoantibodies against ADAMTS13 contribute to the deficiency of ADAMTS13 activity, which leads to the formation of disseminated microvascular platelet thrombi that are attributed to unusually large multimers of von Willebrand factor that cannot be cleaved in the circulation. Acute aortic dissection (AAD) is a cardiovascular emergency that mainly arises from a tear in the aortic intima followed by cleavage formation and spread into the media, although it can also arise from spontaneous bleeding into the vessel wall and hematoma formation.^[2]^ Typical symptoms of AAD include sudden sharp tearing in the chest or back pain, and some patients may have high blood pressure as the initial manifestation. Long-term arterial hypertension, connective tissue disorders, hereditary vascular disease, and vascular inflammation, such as autoimmune diseases, are common risk factors for AAD.^[3,4]^ Here, we describe a patient with acute TTP who developed B aortic dissection during treatment and was discharged after successful thoracic aortic endovascular repair (TEVAR).

## 2 Case report

**Figure 1:**
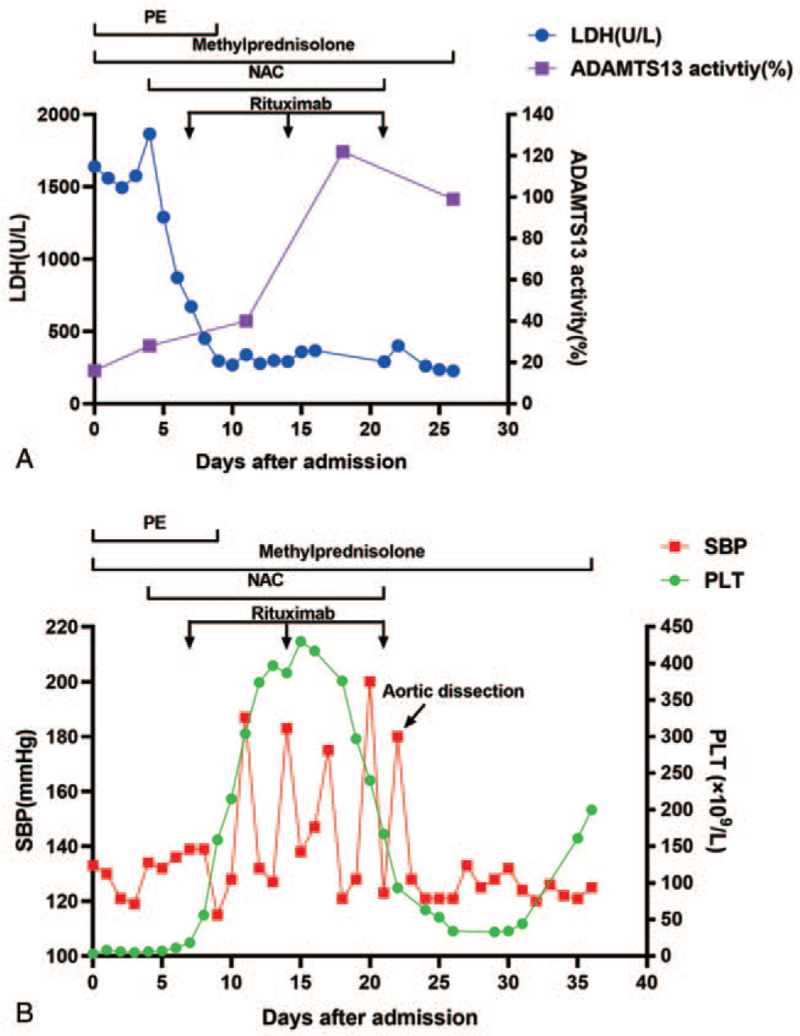
Main treatment and examination results in the clinical course. The treatment included plasma exchange (PE), high-dose methylprednisolone (500 mg/d, gradually reduced to 48 mg/d), rituximab (375 mg/m^2^) and *N*-acetylcysteine (NAC). (A). The trends of ADAMTS13 activity and lactate dehydrogenase (LDH) over the course of treatment, black arrows indicate the 3 rituximab treatments. (B). Changes in systolic blood pressure (SBP) and platelet (PLT) count during the clinical course. The patient underwent aortic dissection on the 22nd day, as indicated by the black arrow.

**Figure 2:**
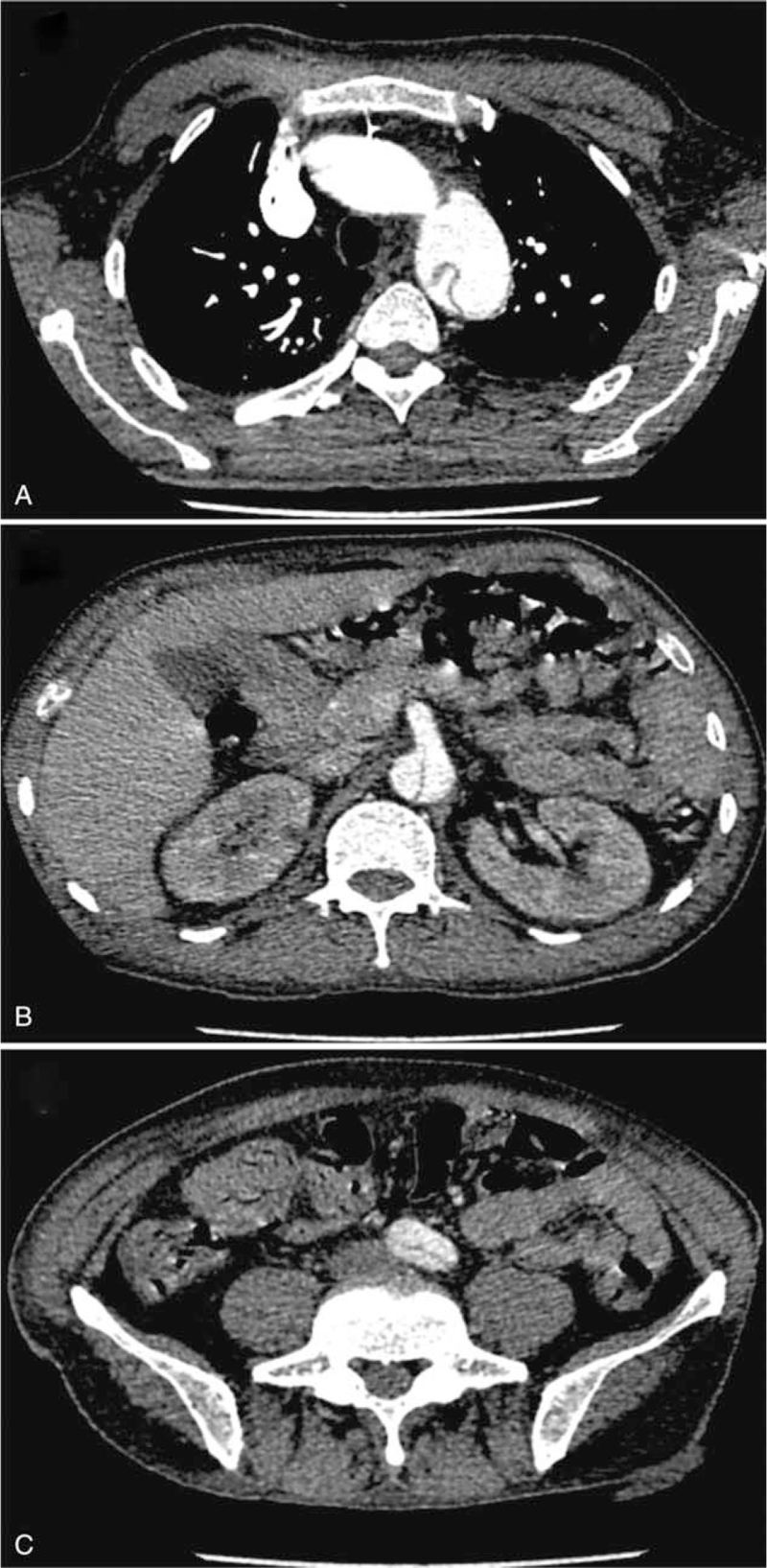
The Chest and abdomen CT angiography (CTA) results of the patient. The CTA showed a Stanford type B aortic dissection, with an incision in the descending thoracic aorta, involving the thoracic aorta (A), abdominal aorta (B), and bilateral common iliac arteries (C).

A 53-year-old Chinese male patient presented to our hospital with ecchymosis and edema for more than 2 months. Routine blood tests showed a hemoglobin level of 62 g/L and platelet count of 10 × 10^9^/L. Suddenly, the patient appeared unconscious and recovered to normal approximately 3 hours later without any obvious abnormality on brain computed tomography (CT) and was then transferred to our hospital. This patient had no special medical history except for left leg trauma 30 years ago, and physical examination showed apathy, pale conjunctiva, and visible skin purpura. Laboratory tests indicated white blood cells 8.37 × 10^9^/L; hemoglobin, 53 g/L; platelet count, 3 × 10^9^/L; reticulocytes, 0.224 × 10^12^/L; total bilirubin 39.7 μmol/L, direct bilirubin 12.0 μmol/L, indirect bilirubin 27.7 μmol/L, lactate dehydrogenase, 1640 U/L, urea 18.75 mmol/L, creatinine 108 μmol/L (normal range 59-104 μmol/L), free hemoglobin (168 mg/L), and haptoglobin < 0.06 g/L. Direct Coombs test results were negative. Rheumatism tests showed that the antinuclear antibody nuclear particle type was 1:100, anti-SSA antibody was 7.1, and anti-SSB antibody was >8.0. Platelet alloantibodies were positive, and platelet autoantibodies were negative. Routine urine tests suggested urinary occult blood 3+, urine protein ±, blood urea 24.82 mmol/L, and creatinine 138 μmol/L. It was easy to see schistocytes on his peripheral blood smear, accounting for 21% of the cells. Bone marrow cytology was characterized by megakaryocytic hyperplasia, maturation disorder, and myeloid hyperplasia. The patient had intermittent fever up to 38°C, intermittent disturbance of consciousness, and transient renal dysfunction during the hospital stay. Based on the above clinical manifestations and laboratory findings, the patient was diagnosed with thrombotic thrombocytopenic purpura with a classic “pentad”, including thrombocytopenia, neurological involvement, microangiopathic hemolytic anemia (elevated indirect bilirubin and reticulocyte, presenting schistocytes on peripheral blood smear), renal impairment and fever, which was likely related to connective tissue disease (CTD). Before measuring the ADAMTS13 activity level, we calculated the PLASMIC score in this patient to predict ADAMTS13 activity.^[5]^ This score includes 7 indicators, with higher scores indicating more likely TTP. The patient had a score of 5 (platelet count <30 × 10^9^ per L, hemolysis variable, no active cancer, no history of solid organ or stem cell transplantation, creatinine <2.0 mg/dL). Before we collected plasma samples for ADAMTS13 activity and inhibitors, he had been transfused with fresh frozen plasma and treated with glucocorticoids. Although the result of ADAMTS13 activity detected by a fluorescence energy transfer assay was 16%, experts with experience in management of TTP still believed that the patient should be diagnosed with TTP because of typical clinical manifestations and ADAMTS13 inhibitor positivity.^[6]^ He was treated with daily plasma exchange (9 times) at 1.0× patient plasma volume until the platelet count recovered to normal and was maintained for at least 2 days, and his clinical symptoms were relieved. In addition, the patient received high-dose glucocorticoids (methylprednisolone 500 mg/d) for 5 days, which was then gradually reduced to 48 mg/d; *N*-acetylcysteine for 17 days, and 3 once-weekly doses of rituximab (375 mg/m^2^) (Fig. 1A). Lactate dehydrogenase gradually decreased, and ADAMTS13 activity gradually increased (Fig. 1A). Her platelet count returned to normal (Fig. 1B). On the 11th day after admission, the patient's blood pressure suddenly increased to 187/98 mm Hg, mainly at night and in the morning, without any other discomfort (Fig. 1B). Ambulatory blood pressure monitoring revealed high blood pressure and increased pulse pressure at night, accompanied by the disappearance of the circadian rhythm, while the adrenal enhanced CT scan showed no obvious abnormalities. On the 22nd day after admission, the patient experienced sudden chest pain with blood pressure up to 180/85 mm Hg. Chest and abdominal computed tomography angiography (CTA) was performed immediately, which suggested the existence of an AAD, with an incision in the descending thoracic aorta, involving the thoracic aorta (Fig. 2A), abdominal aorta (Fig. 2B), and bilateral common iliac arteries (Fig. 2C), confirming the appearance of acute type B aortic dissection. Subsequently, the patient was transferred to the cardiac aortic surgery department for TEVAR and discharged 13 days after the operation when the platelet count returned to normal, despite a transient decrease in platelet count that occurred during the aortic dissection. The platelet count remained stable after the patient was discharged. However, fever and pulmonary infections occurred later. Because the patient lived far away from our hospital, he underwent reexamination and anti-infection treatment at the local hospital. Unfortunately, 3 months after the operation, the patient experienced chest and back pain at home and died suddenly, which may have been caused by recurrence of the dissection.

## 3 Discussion

To our knowledge, this is the first report of TTP complicated by AAD. It is difficult to clarify the underlying mechanisms of AAD in patients without risk factors. Based on the abnormal antinuclear antibody (nuclear particle type) 1:100 and increased anti-SSA antibody that we observed, we speculated that it may be associated with underlying CTD. The episodes of acquired TTP are often accompanied by or triggered by other diseases, including autoimmune disorders, drugs, cancer, surgery, infection, transplantation, and pregnancy.^[7,8]^ The presence of autoantibodies against ADAMTS13 reveals immune dysfunction in patients with acquired TTP.^[7]^ A variety of CTDs that are reported to be concurrent or secondary to TTP are also related to autoimmune diseases, including systemic lupus erythematosus, mixed connective tissue disease, and systemic scleroderma.^[9–11]^ It is estimated that 9% of patients with TTP have a medical history of CTD, and 21.5% of patients with TTP may have preexisting, co-existing, or subsequent autoimmune diseases. The appearance of anti-SSA antibodies or anti-dsDNA antibodies at TTP diagnosis has been closely related to autoimmune disorders during follow-up.^[12]^ Moreover, fragmentation of elastic fibers and disturbance of smooth muscle cells in the aortic intima of patients with CTD lead to an abnormal mechanical structure of the aortic wall and increased fragility of the vessel wall.^[13]^ Vascular damage caused by various connective tissue diseases further leads to aortic dissection or other vascular diseases.

When patients present with thrombocytopenia and microangiopathic hemolysis, TTP should be suspected, and daily plasma exchange should be performed immediately until a clinical response is obtained.^[14]^ Glucocorticoids are also recommended. However, long-term use of glucocorticoids may lead to cardiovascular complications, accelerating atherosclerosis and resulting in aortic aneurysmal enlargement, especially in the context of potential vasculitis and vascular involvement in CTD or TTP.^[15]^ This patient had no cardiovascular or endocrine disease, and his blood pressure was normal upon admission. However, the patient had an intermittent nocturnal blood pressure increase before AAD, which may be related to glucocorticoid effects or early manifestations of aortic dissection. Hemodynamic abnormalities further promote the occurrence of aortic dissection on the existing structural abnormalities of the arterial wall. In addition, patients with thrombotic thrombocytopenia may have a tendency for spontaneous hemorrhage in the aorta. USUI and Tsai reported a patient with AAD with idiopathic thrombocytopenic purpura, neither of whom had risk factors for dissection, suggesting that dissection may be associated with an increased risk of spontaneous bleeding in the aortic wall caused by thrombocytopenia.^[16,17]^ However, transient thrombocytopenia in this patient before AAD may be ascribed to consumptive bleeding initiated by AAD because the patient's platelet count remained normal for many days and quickly returned to normal after the operation.

Imaging examinations are essential not only to diagnose AAD in a timely manner but also to display the localization of tears, determine the type of AAD, and guide therapeutic intervention. Noninvasive CT and contrast-enhanced CTA are the most commonly used methods. According to the Stanford system, aortic dissection is divided into 2 types: acute type A dissection, involving the ascending aorta, requires urgent surgical treatment, because the mortality rate increases by 1% to 2% per hour after the onset of symptoms, and if untreated, 50% of patients die within 1 week, Stanford type B mainly involves the descending aorta at the distal end of the left subclavian artery, and medical management remains the preferred treatment option for uncomplicated acute type B dissection.^[3]^ TEVAR to eliminate the false lumen and ensure true lumen by stent-grafting to seal the rupture is recommended for complicated acute type B aortic dissection.^[18]^ However, some authors believe that prophylactic repair of acute type B dissection can also benefit high-risk patients and prevent late complications.^[18–21]^ Once diagnosed with Stanford type B aortic dissection, the patient was immediately transferred to the cardiac aortic surgery department for TEVAR treatment.

## 4 Conclusion

In conclusion, early diagnosis of AAD in TTP remains a challenge for clinicians. We describe the first case of TTP complicated with acute dissection type B that occurred during TTP treatment, hoping to raise awareness among clinicians about this rare complication. When back pain or thoracoabdominal pain and unexplained hypertension occur in patients with TTP, aortic dissection should be suspected, and CTA examination should be performed promptly. It is necessary to monitor blood pressure closely and control it in a timely manner during glucocorticoid treatment. Whether TTP is a risk factor for spontaneous bleeding within the aorta and the relationship between TTP and AAD is still worthy of further discussion.

## Author contributions

**Conceptualization:** Jin-Niu Deng.

**Supervision:** Jian-Feng Zhou.

**Writing – original draft:** Mei-Juan Huang.

**Writing – review & editing:** Li-Li Gao.
